# Dietary patterns and quality of life among night-shift nurses in tertiary hospitals in Hangzhou: a cross-sectional analysis

**DOI:** 10.3389/fpubh.2025.1638082

**Published:** 2025-10-20

**Authors:** Guanmian Liang, Rongyu Hua, Fangying Yang

**Affiliations:** ^1^Nursing Department, Zhejiang Cancer Hospital, Hangzhou, Zhejiang, China; ^2^Gastroenterology Surgery Ward, Zhejiang Cancer Hospital, Hangzhou, Zhejiang, China

**Keywords:** dietary pattern, nurse, quality of life, SF-36, night-shift work

## Abstract

**Objective:**

This study aimed to examine the dietary patterns of nurses working night shifts in tertiary hospitals in Hangzhou and to assess the association between these patterns and quality of life. The objective is to provide evidence from a nutritional standpoint to inform health promotion strategies within the nursing workforce.

**Method:**

A cross-sectional design was employed. A total of 1,024 valid questionnaires of dietary intake data were collected using the simplified Food Frequency Questionnaire (FFQ25), and quality of life was assessed via the 36-Item Short Form Survey (SF-36). Factor analysis was utilized to identify major dietary patterns. One-way analysis of variance was conducted to explore differences in quality-of-life scores across dietary pattern groups.

**Results:**

Three primary dietary patterns were identified: Traditional, Western, and Balanced. The traditional and balanced dietary patterns were associated with higher SF-36 scores across most dimensions compared to the western dietary pattern, with the exception of the general health dimension. Statistically significant differences were observed between the Traditional and Western patterns in physical functioning, emotional state, health transition, and general health (*p* < 0.05). Comparisons between the Balanced and Western patterns revealed significant differences in general health and health transition (*p* < 0.05), with no significant differences in other domains.

**Conclusion:**

Distinct dietary patterns were observed among nurses engaged in night-shift work, with associations noted between specific patterns and quality-of-life outcomes. These findings suggest that dietary behavior may serve as a proxy for broader health-related behaviors. Interventions targeting nutritional habits may contribute to enhanced quality of life and support comprehensive health promotion strategies among nursing personnel.

## 1 Introduction

As part of the National Quality Nursing Service Demonstration Project, the National Health and Family Planning Commission emphasized the importance of supporting nurses’ quality of life and safeguarding their physical and mental well-being, along with protecting their legitimate rights and interests. To maintain the quality and safety of nursing care, shift work, particularly night-shift rotations, has been widely implemented in clinical settings. However, night-shift rotations have been associated with circadian rhythm disturbances, which may increase the risk of various adverse health outcomes, including cancer and metabolic syndrome, as well as negatively influence mental health and cognitive function among rotating shift workers. It should be clarified that the aforementioned associations between night-shift work and risks of cancer or metabolic syndrome are all based on existing observational studies and meta-analyses, rather than representing direct causal relationships (1–6).

Specifically, studies on the association between night-shift work and cancer risk have provided evidence for this link: a meta-analysis of 16 prospective cohort studies demonstrated that night-shift work increases the morbidity of breast cancer and all-cause mortality (1), and another investigation combining three prospective studies with a meta-analysis of published literature further explored the relationship between night-shift work and breast cancer incidence (2). Additionally, research on circadian rhythm and sleep patterns has laid a theoretical foundation for understanding how night-shift work may contribute to cancer risk by disrupting physiological rhythms (3). Regarding the association between night-shift work and metabolic syndrome: a meta-analysis explicitly confirmed that night-shift work is associated with an elevated risk of metabolic syndrome (4); another study focusing on night-shift healthcare workers found a notable incidence of metabolic syndrome in this population (5); and results from two large U.S. cohorts of female nurses revealed that rotating night-shift work, combined with adherence to an unhealthy lifestyle, predicts the risk of type 2 diabetes—an important component of metabolic syndrome (6). Furthermore, other studies have explored factors related to mental health and cognitive function in shift workers: one study examined the roles of shift work disorder, depression, anxiety, and sleep reactivity during the transition to rotating shifts (7), while another reviewed the mechanisms by which disrupted sleep affects physical and cognitive functions from molecular to behavioral levels (8).

Notably, factors such as age, duration of night-shift work, sleep quality, and exercise frequency may modulate the associations between night-shift work and disease risks. These modulating factors need to be considered in future research to more accurately assess the actual impact of night-shift work on nurses’ health. These challenges are particularly pronounced in tertiary public hospitals, where the clinical environment is more demanding compared to lower-tier healthcare institutions due to higher patient volumes, increased case complexity, and the critical condition of many hospitalized patients (9). The “2023 National Medical Quality Report on Tertiary Hospitals in China” released by the National Health Commission (NHC) of the People’s Republic of China in 2023 points out that “the volume of nighttime emergency visits in tertiary hospitals accounts for 30–35% of the total daily emergency volume, and the difficulty score of cases is 20–25% higher than that in secondary hospitals” (10). Meanwhile, the survey results of a 2025 study titled “Fatigue Status and Coping Strategies of Night-Shift Nurses in China: A Cross-Sectional Study” show that “night-shift nurses in tertiary hospitals are responsible for 8–10 patients per person on average, which is significantly higher than the 5–6 patients per person in secondary hospitals; the proportion of critically ill patients is 35–40%, more than twice that of secondary hospitals” (11).

The resulting workload places considerable stress on nursing staff. In addition to circadian rhythm disruptions and occupational stressors, dietary habits during night shifts represent a significant factor influencing quality of life among nurses (12). Specifically, Zhang et al. (13) pointed out that night-shift nurses, due to the dietary decision-making chain of “circadian rhythm disruption—delayed hunger - priority to convenience,” have a frequency of high-oil and high-sugar food intake (4–5 times per week), which is 1.8 times that of day-shift nurses. In terms of the association between dietary patterns and quality of life, Chiang et al. (14) found that the dietary habits of night-shift nurses were inferior to those of fixed-shift nurses, and their quality of life was also lower than that of fixed-shift nurses.

For instance, adherence to the Mediterranean dietary pattern, a model recognized for its health benefits, has been associated with improvements in quality of life (15). Although the effects of diet on quality of life have been extensively studied in the general population, research specifically on night-shift nurses in tertiary hospitals remains very limited. More importantly, existing studies have mostly explored the associations between “night shifts and diet” or “diet and quality of life” separately, while there is a paucity of research focusing on the chain association of “night shifts - dietary patterns - quality of life.” This research gap highlights the necessity of addressing this understudied area, and the present study intends to fill this void.

Therefore, the present study aims to analyze the association between specific dimensions of dietary patterns (e.g., frequency of vegetable and fruit intake, proportion of high-quality protein, frequency of high-sugar and high-fat food consumption) and specific dimensions of quality of life (i.e., physical function, physical pain, vitality, social function, psychological function, and general health) among night-shift nurses in a tertiary hospital in Hangzhou, China. It further seeks to clarify the differences in the impacts of different dietary patterns on each dimension of night-shift nurses’ quality of life, thereby providing a targeted scientific basis for developing health intervention strategies.

## 2 Participants and methods

### 2.1 Participants

Between June and December 2024, clinical nurses employed at tertiary grade-A hospitals in Hangzhou were recruited through convenience sampling. Regarding the sampling method, it is important to note that while this convenience sampling approach covered over 90% of night-shift nurses in the included hospitals, selection bias remains unavoidable. As a result, the study findings are only generalizable to night-shift nurses in the participating hospitals and other tertiary hospitals of similar scale; caution should be exercised when extending these results to other populations. The inclusion criteria were as follows: being a registered nurse; engaging in clinical practice; having a minimum of 1 year of work experience; currently being employed at a tertiary grade-A hospital in Hangzhou; and participating in rotating night-shift schedules. Nurses who had not performed night-shift work for 3 months or more were excluded. All participants provided informed consent and voluntarily agreed to participate in the study.

### 2.2 Methods

#### 2.2.1 Survey instruments

##### 2.2.1.1 General information questionnaire

A self-designed questionnaire was used to collect demographic and occupational information. This included sex, age, years of work experience, department, night shift type, number of night shifts per month, height, weight, marital status, and parental status.

##### 2.2.1.2 Simplified food frequency questionnaire

The Food Frequency Questionnaire (FFQ25) was employed to assess dietary intake over the preceding month. Originally developed and validated in 2011 by Dr. Gao Jian at Fudan University, the FFQ25 has demonstrated acceptable test–retest reliability and construct validity in dietary pattern assessments (16). It is noted for its simplicity and ease of use. The questionnaire consists of 50 items encompassing 25 food categories, including rice, porridge, flour-based foods, sweets, fried foods, stuffed foods, whole grains, tubers, milk, eggs, red meat, poultry, processed meats, freshwater and marine seafood, soy products, nuts, dark- and light-colored vegetables, mushrooms, fruits, sugary beverages, beer, yellow rice wine, and white spirits. Dietary patterns were identified through exploratory factor analysis. Prior to this, the Kaiser-Meyer-Olkin (KMO) test and Bartlett’s test of sphericity were conducted to assess sampling adequacy and factorability. When classifying dietary patterns, ‘reverse scoring’ is adopted for foods with negative factor loadings - for example, for ‘beer intake frequency’: ‘once a day’ is scored 1 point, ‘once a week’ is scored 3 points, and ‘once a month or less’ is scored 5 points; ‘positive scoring’ is used for foods with positive factor loadings (such as vegetables, fruits, and fish). Finally, based on the ‘total score of all foods’, the research subjects are divided into ‘low dietary pattern group (1–33 points), medium dietary pattern group (34–66 points), and high dietary pattern group (67–100 points)’.

##### 2.2.1.3 The medical outcomes study short form health survey

The 36-Item Short Form Survey (SF-36) is a standardized health survey instrument developed from the Medical Outcomes Study Short Form (MOS SF) by Stewart et al. in 1988 and subsequently refined by the Health Institute at Boston. In 1991, the Department of Social Medicine at Zhejiang University School of Medicine translated and culturally adapted the Chinese version, which has demonstrated acceptable reliability and validity for use in the general population. Its applicability to healthcare professionals was further supported by validation studies conducted by Tang et al. (17). The SF-36 comprises 36 items that assess eight dimensions of health-related quality of life: physical functioning (PF), role limitations due to physical problems (RP), bodily pain (BP), general health (GH), vitality (VT), social functioning (SF), role limitations due to emotional problems (RE), and mental health (MH). Additionally, one item evaluates health transition (HT), reflecting perceived changes in overall health over the previous year. Each dimension is scored on a scale ranging from 0 to 100, with higher scores indicating better perceived quality of life. Scoring procedures followed the standard methodology outlined in prior research, and scores were calculated for all dimensions, including PF, RP, BP, GH, VT, SF, RE, MH, and HT (17).

#### 2.2.2 Survey procedures

Prior to survey administration, approval and support were obtained from hospital administrators. The hospital nursing departments coordinated the distribution of questionnaires, which were disseminated via the online platform “Wenjuanxing.” We set an electronic informed consent form at the beginning of the questionnaire link. Participants must check the “Agree” option to enter the questionnaire page. To ensure that the research subjects meet the inclusion criteria, we screened the list of night shift nurses in advance based on the nursing department schedule to ensure the accuracy of the survey subjects. Departmental head nurses supervised the process to enhance response accuracy and data integrity. To prevent duplicate submissions, the platform was configured to allow only one entry per IP address. And when submitting, use the “mobile phone verification code” for verification (the mobile phone number is provided by the head nurse to ensure only the target nurse participates), to avoid “repeated filling due to shared network or filling by non-target personnel.” The FFQ25 included color reference images of food portion sizes to support participants in estimating their intake more accurately. In terms of weight measurement, all participants are required to provide self-testing data within 1 month. Following data collection, responses were reviewed. For questionnaires without responses, we increased the response rate through the method of “reminders from the head nurse and two follow-up calls from the researcher.” Regarding the issue of missing data, in the early stage of the questionnaire, we set “required options” for relatively core data such as age and dietary frequency. If these were not filled in, the questionnaire could not be submitted, which was used to control the quality of the questionnaire filling. At the same time, questionnaires with a filling time of less than 5 min (the average filling time of the questionnaire was 12 min) and those with logical contradictions (such as “night shift working years > total working years”) were marked as invalid and excluded from the analysis.

### 2.3 Sample size justification

The target sample size was determined *a priori* based on the requirements for conducting factor analysis, the primary method for identifying dietary patterns. A widely accepted convention for factor analysis recommends a minimum subject-to-variable ratio of 10:1. Given that FFQ comprised 25 food items, a minimum sample of 250 participants was required. We distributed approximately 1,100 questionnaires to achieve a final sample size well above this threshold. Our final analytical cohort of 1,024 participants provided ample statistical power for the factor analysis and subsequent analyses, minimizing the risk of overfitting and ensuring the generalizability of the results.

### 2.4 Statistical analysis

All statistical analyses were conducted using SPSS version 20.0. The normality of distribution for all continuous variables was assessed. In this study, the Shapiro–Wilk test was selected to assess the normality of continuous variables based on the sample size (*n* = 1,024). The results showed that the W values for age (*W* = 0.96, *p* = 0.12), BMI (*W* = 0.95, *p* = 0.08), and the scores of each dimension of SF-36 (*W* = 0.94–0.97, *p* > 0.05) were all greater than 0.90, and the *p* values were all greater than 0.05, indicating an approximate normal distribution. Therefore, parametric tests such as t-tests and one-way ANOVA were used in subsequent analyses. Continuous variables were expressed as mean ± standard deviation, while categorical variables were summarized using composition ratios. Between-group comparisons for continuous variables were performed using t-tests or one-way analysis of variance, while categorical variables were compared using the chi-squared (*χ*^2^) test. Dietary patterns were extracted using exploratory factor analysis with principal component extraction. Factors with eigenvalues greater than 1 were retained. The KMO measure and Bartlett’s test of sphericity were conducted prior to factor analysis to confirm the adequacy of the data. Each individual’s factor scores were calculated, and classification into a dietary pattern group was based on the highest factor score. A two-tailed *p*-value < 0.05 was considered statistically significant.

### 2.5 Ethics

This study has been approved by the Ethics Review Committee of Zhejiang Cancer Hospital, with the approval number IRB-2023-1103 (IIT). All procedures comply with the relevant requirements of the Declaration of Helsinki.” The questionnaire data is only used for statistical analysis in this study and is stored on an encrypted server (the password is kept separately by two researchers). After the study is completed, it will be preserved for 3 years in accordance with the “Medical Research Data Management Standards,” and then completely destroyed upon expiration to ensure that the personal information of the participants is not disclosed.

## 3 Results

### 3.1 Basic characteristics of the study participants

A total of 1,100 questionnaires were collected. After excluding 76 questionnaires due to missing responses, logical inconsistencies, incorrect answers, or failure to meet the inclusion criteria, 1,024 valid questionnaires remained, yielding an effective response rate of 94%. Among the participants, 25 (2.4%) were male and 999 (97.6%) were female. The mean body mass index (BMI) was 21.37 kg/m^2^. Detailed demographic and work-related characteristics are presented in Table 1.

**Table 1:** Basic characteristics of clinical nurses participating in the study (*N* = 1,024).

Characteristic	Group	Number (n)	Percentage (%)
Age (years)	18–25	201	19.6
	26–30	294	28.7
	31–40	431	42.1
	41–50	83	8.1
	51~	15	1.5
Sex	Male	25	2.4
	Female	999	97.6
Marital status	Unmarried	346	33.8
	Married	649	63.4
	Cohabiting	10	1.0
	Divorced or Widowed	19	1.9
Children	Yes	591	57.7
	No	433	42.3
Title	Nurse	212	20.7
	Nurse practitioner	453	44.2
	Head nurse	335	32.7
	Director or deputy director of nursing	24	2.3
Department	Internal medicine	539	52.6
	Surgery	271	26.5
	Special departments*	214	20.9
Years of work experience	<5	305	29.8
	5–10	307	30.0
	10–15	210	20.5
	15–20	92	9.0
	≧20	110	10.7

*Special departments include: ICU, operating room, emergency, delivery room, and monitoring room.

### 3.2 Dietary patterns among clinical nurses

Sampling adequacy tests indicated that the data were suitable for factor analysis. The KMO measure was 0.764 (> 0.6), and Bartlett’s test of sphericity was statistically significant (*p* < 0.001), supporting the appropriateness of factor analysis for the intake data across 25 food categories.

Exploratory factor analysis identified nine components with eigenvalues greater than 1. Based on eigenvalues, the number of components preceding the inflection point on the screen plot, and interpretability, three principal dietary patterns were retained. These three factors had eigenvalues of 3.20, 2.46, and 2.35, accounting for 12.80, 9.83, and 9.39% of the variance, respectively, with a cumulative variance contribution rate of 32.01%. Factor loadings are presented in Table 2. The first dietary pattern, labeled the traditional dietary pattern, was primarily characterized by tubers and whole grains, with supplementary intake of fish, vegetables, soy products, and eggs. The second pattern, termed the western dietary pattern, demonstrated higher consumption of fried foods, processed meats, flour-based foods, and stuffed foods, along with sweets, nuts, and sugary beverages. The third pattern, identified as the balanced dietary pattern, reflected a more evenly distributed intake of vegetables, fruits, meat, eggs, and dairy products.

**Table 2:** Factor loadings for identified dietary patterns among clinical nurses.

Traditional dietary pattern	Factor loadings	Western dietary pattern	Factor loadings	Balanced dietary pattern	Factor loadings
Tubers	0.71	Fried food	0.69	Dark-colored vegetables	0.59
Whole grains	0.70	Processed meats	0.57	Fruits	0.55
Seafood	0.63	Flour-based food	0.50	Light-colored vegetables	0.84
Mushrooms	0.54	Stuffed food	0.47	Red meats	0.44
Freshwater seafood	0.53	Sugary beverages	0.45	Eggs	0.42
Soy products	0.53	Red meats	0.34	Poultry	0.41
Sweets	0.43	Rice	0.33	Milk	0.39
Nuts	0.35	Nuts	0.32	Beer	−0.37
Light-colored vegetables	0.39	Seafood	0.32	Yellow rice wine	−0.33
Eggs	0.38	Freshwater aquatic foods	0.31	White spirits	−0.31
		Sweets	0.30		

Only foods or food groups with absolute factor loadings ≥ 0.30 are listed. And negative factor loading meaning: The intake frequency of this type of food (such as beer, yellow wine, and white wine) is negatively correlated with the ‘balanced diet pattern’, that is, the more consumed, the less it conforms to the ‘balanced diet’ characteristics.

### 3.3 Comparison of basic characteristics across dietary patterns

One-way analysis of variance indicated that there were no statistically significant differences among the low, medium, and high dietary pattern groups in terms of demographic characteristics such as age, gender, years of work, and marital status, as well as work-related characteristics such as “number of night shifts per week” and “years of night shift work” (*p* > 0.05). Detailed comparisons are presented in Table 3.

**Table 3:** Comparison of demographic and work-related characteristics across dietary patterns.

Characteristic	Different dietary patterns			*χ^2^/F*	*p*
	Traditional dietary pattern (*n* = 301)	Western dietary pattern (*n* = 334)	Balanced dietary pattern (*n* = 389)		
Age (years)				4.878	0.899
18–25	56	65	80		
26–30	90	96	108		
31–40	122	145	164		
41–50	29	22	32		
51–60	4	5	5		
>60	0	1	0		
Sex				3.693	0.158
Male	6	5	14		
Female	295	329	375		
Marital status				9.081	0.169
Unmarried	94	112	140		
Married	197	213	239		
Cohabiting	1	6	3		
Divorced or Widowed	9	3	7		
Children				5.403	0.067
Yes	189	192	210		
No	112	142	179		
Title				6.875	0.333
Nurse	53	68	91		
Nurse practitioner	142	149	162		
Head nurse	101	111	123		
Director or deputy director of nursing	5	6	13		
Department				3.059	0.548
Internal medicine	148	180	211		
Surgery	87	81	103		
Special departments*	66	73	75		
Years of work experience				8.820	0.358
<5	82	100	123		
5–10	88	99	120		
10–15	68	78	64		
15–20	26	27	39		
≧20	37	30	43		
BMI (kg/㎡)	21.09 ± 2.43	21.35 ± 3.25	21.09 ± 2.91	0.896	0.408

BMI is described as x̄ ± standard deviation (±s), statistical test is *F* value; other variables are described as counts (*n*), statistical test is *χ*^2^ value. *Special departments include: ICU, operating room, emergency, delivery room, and monitoring room.

### 3.4 Association between dietary patterns and quality of life

Each participant was assigned to a dietary pattern group based on the highest individual factor score. Of the 1,024 participants, 301 (29.4%) were classified under the traditional dietary pattern, 334 (32.6%) under the western dietary pattern, and 389 (38.0%) under the balanced dietary pattern. SF-36 scores indicated that the PF dimension had the highest mean scores across all groups, with the balanced dietary pattern group reporting the highest PF score (85.78 ± 12.72). The HT dimension had the lowest scores, with the western dietary pattern group recording the lowest HT score (31.14 ± 20.04).

Compared to the western dietary pattern, participants in the traditional dietary pattern group had significantly higher scores in the RP, RE, and HT dimensions (*p* < 0.05), but lower scores in the GH dimension (*p* < 0.05). No significant differences were observed in the BP, VT, SF, or MH dimensions.

No statistically significant differences were observed between the Traditional and balanced dietary pattern groups across any SF-36 dimensions (*p* > 0.05).

Compared with the western dietary pattern group, the balanced dietary pattern group scored significantly higher in the HT dimension (*p* < 0.05) but significantly lower in the GH dimension (*p* < 0.05). No significant differences were found in the remaining dimensions. Detailed results are presented in Table 4.

**Table 4:** Comparison of SF-36 quality of life scores among clinical nurses by dietary pattern (*N* = 1,024).

SF-36 scale	Different dietary patterns			*p*
	Traditional dietary pattern (*n* = 301)	Western dietary pattern (*n* = 334)	Balanced dietary pattern (*n* = 389)	
PF	85.55 ± 13.14	84.51 ± 12.87	85.78 ± 12.72	0.382
RP	60.05 ± 36.18^a^	53.89 ± 37.32	56.49 ± 37.62	0.113
BP	62.48 ± 15.56	63.07 ± 15.05	61.72 ± 16.19	0.507
GH	65.03 ± 20.52^a^	68.86 ± 20.04^b^	65.75 ± 20.49	0.037^*^
VT	64.73 ± 16.42	62.66 ± 15.88	63.66 ± 15.37	0.278
SF	64.49 ± 13.70	63.51 ± 13.85	62.66 ± 15.37	0.253
RE	57.36 ± 36.95^a^	50.40 ± 38.29	54.50 ± 37.00	0.061
MH	61.62 ± 16.33	60.22 ± 16.28	60.37 ± 17.43	0.512
HT	34.96 ± 20.52^a^	31.14 ± 20.04^b^	34.25 ± 20.49	0.037^*^

PF, physical functioning; RP, role limitations due to physical problems; BP, bodily pain; GH, general health; VT, vitality; SF, social functioning; RE, role limitations due to emotional problems; MH, mental health; HT, health transition. *p < 0.05.

a Comparison between traditional dietary pattern and western dietary pattern, *p* < 0.05.

b Comparison between western dietary pattern and balanced dietary pattern, *p* < 0.05.

## 4 Discussion

### 4.1 Distinct dietary patterns among night-shift nurses in tertiary hospitals in Hangzhou

In the present study, dietary patterns were identified and named based on the characteristic composition of food items, with a factor loading threshold of > 0.30 considered indicative of a strong association between a food item and a given dietary pattern (15). Three primary dietary patterns were extracted—traditional, western, and balanced—accounting collectively for 32.01% of the total variance in food intake.

The traditional dietary pattern was characterized by high consumption of seafood and a base of grains and whole grains, supplemented with vegetables, fruits, and soy products. This pattern aligns with regional dietary practices common in southern China, particularly in the Jiangnan region, where Hangzhou is located. The dietary habits identified here reflect local cultural and culinary preferences.

The western dietary pattern was marked by increased intake of high-fat, energy-dense foods, including fried foods, processed meats, flour-based items, stuffed foods, and sugary beverages, with a notable emphasis on meat. This pattern may be prevalent during night shifts due to the availability of such foods at 24-h food outlets. Previous research has indicated that limited rest, inadequate food options in hospital cafeterias, and restricted cafeteria operating hours contribute to the adoption of Western dietary habits among nurses (18). These findings underscore the importance of modifying the workplace food environment to support healthier eating behaviors among healthcare staff (19). The balanced dietary pattern included a broad intake of vegetables and fruits, along with a balanced proportion of both plant- and animal-based food sources, thereby providing comprehensive nutritional coverage. This pattern is generally regarded as a healthy dietary structure.

No statistically significant differences were observed in marital status, parental status, sex, professional title, or departmental affiliation across dietary pattern groups. These findings indicate that such sociodemographic and occupational variables did not significantly influence dietary behavior in this population. While existing literature has indicated that socioeconomic status may affect dietary choices, the relatively uniform socioeconomic background of clinical nurses employed in tertiary grade A hospitals in Hangzhou may have limited such variation in this study (20). Meanwhile in this study the sample size of male night shift nurses is relatively small, the *χ*^2^ test results may have insufficient power, and subsequent studies can expand the sample size of male night shift nurses to verify the conclusion.

### 4.2 Association between dietary patterns and quality of life

A comprehensive assessment of dietary patterns among clinical nurses facilitates a more nuanced understanding of the relationship between nutritional habits and quality of life.

#### 4.2.1 Nurses following traditional or balanced dietary patterns tend to report better quality of life

Nurses adhering to Traditional or balanced dietary patterns demonstrated higher quality of life scores in comparison to those following a western dietary pattern, particularly in the SF-36 dimensions of RP, RE, and HT. The RP and RE dimensions are reflective of functional limitations attributed to physical and emotional health conditions. Findings from qualitative research have indicated that nurses perceive healthy dietary practices as beneficial for both psychological and physiological well-being (21). Participants in such studies reported that adopting healthy eating habits contributed to reduced stress, greater energy levels, and overall improved physical condition.

Components of the traditional and balanced dietary patterns, such as whole grains, tubers, vegetables, and fruits, are known to be rich in dietary fiber, which has been associated with reduced systemic inflammation, improved gastrointestinal function, and lower cardiovascular risk (22). The inclusion of seafood provides n-3 fatty acids, which offer protective effects against cardiovascular and cerebrovascular conditions, as well as certain cancers. Furthermore, soy products and eggs contribute to sufficient dietary protein intake. Overall, both Traditional and balanced dietary patterns were characterized by dietary diversity and nutritional adequacy, indicating their potential role in promoting long-term improvements in quality of life. Specifically, the mean HT scores over the past year were higher in the traditional dietary pattern group (34.96 ± 20.52) and the balanced dietary pattern group (34.25 ± 20.49) compared to the western dietary pattern group (31.14 ± 20.04).

### 4.3 Night-shift nurses following a western dietary pattern may be at increased risk of health complications

Night-shift clinical nurses adhering to a western dietary pattern exhibited lower overall quality of life, as indicated by a total SF-36 score of 126.78 ± 24.03. This was lower than the scores observed among individuals following the traditional dietary pattern (130.33 ± 23.84) and the balanced dietary pattern (127.73 ± 24.71). The western dietary pattern—characterized by the frequent consumption of high-fat and high-calorie foods—when combined with the physiological disruption associated with night-shift schedules, may elevate the risk of obesity and related adverse health outcomes. Evidence has indicated that eating during biologically inappropriate times in the circadian cycle, such as nighttime, constitutes a novel risk factor for weight gain and obesity (23). This pattern may partly explain the lower self-rated HT scores observed among participants following the western dietary pattern.

Despite these findings, individuals in the western dietary pattern group reported higher GH scores (68.86 ± 20.04) than the national population norm (66.03 ± 20.87) (24). The GH domain reflects subjective health evaluations in areas such as perceived susceptibility to illness, comparisons with peers, perceived health decline, and self-assessed health excellence. It is plausible that individuals in this group, owing to their clinical training and familiarity with medical conditions, possess enhanced awareness and confidence in evaluating their own health, which may contribute to inflated GH scores. Notably, the GH score for the western dietary pattern group exceeded those of both the traditional and balanced dietary pattern groups. One possible explanation is that nurses may use food consumption as a coping mechanism to manage occupational stress during night shifts. Due to altered meal schedules and limited food availability at night, many hospital cafeterias remain closed, leaving fast food as the most accessible option. These foods typically include fried items, processed meats, sugary beverages, and desserts—hallmarks of the western dietary pattern. Furthermore, studies have indicated that severe sleep restriction significantly increases the likelihood of snacking, with a marked preference for desserts under such conditions (25). These findings support the notion that consumption of Western-style foods during night shifts may offer short-term psychological comfort or stress relief.

Although a temporary sense of well-being may be derived from this eating pattern, the cumulative intake of energy-dense, nutrient-poor foods, combined with irregular sleep and dietary routines, imposes long-term physiological strain. These behaviors may ultimately contribute to deteriorating health and diminished quality of life over time. Accordingly, efforts should be directed toward reducing the intake of foods typical of the western dietary pattern to mitigate long-term health risks among night-shift clinical nurses.

### 4.4 A Balanced Diet Pattern may have a positive impact on the multi-dimensional quality of life of night shift nurses

Integrating the scores of Physical Function (PF, *p* = 0.382), Bodily Pain (BP, *p* = 0.507), Vitality (VT, *p* = 0.278), Social Function (SF, *p* = 0.253), and Mental Health (MH, *p* = 0.512) in Table 4 and conducting trend analysis based on night shift nurses’ characteristics (long-term physical exertion, night work rhythms, occupational social requirements), it should be noted that the lack of statistical significance for these results may stem from limited sample size, and future research could verify such trends by expanding sample size; specifically, PF scores of traditional (85.55 ± 13.14) and balanced (85.78 ± 12.72) dietary patterns were comparable and slightly higher than Westernized (84.51 ± 12.87), implying potential support for muscle endurance and physical coordination (relevant to patient transfer and long standing); BP scores of Westernized pattern (63.07 ± 15.05) were marginally higher than traditional (62.48 ± 15.56) and balanced (61.72 ± 16.19) (higher BP scores mean less pain interference), suggesting subtle pain-relieving effects of components like Omega-3 fatty acids; VT scores showed “traditional (64.73 ± 16.42) > balanced (63.66 ± 15.37) > Westernized (62.66 ± 15.88),” possibly related to slow energy release from complex carbohydrates in traditional diets (supporting nocturnal energy supply); SF scores of traditional pattern (64.49 ± 13.70) were slightly higher than Westernized (63.51 ± 13.85) and balanced (62.66 ± 15.37), potentially linked to family meals and regular eating habits (aiding interpersonal interaction); while MH scores showed no obvious trend across the three patterns (traditional: 61.62 ± 16.33; Westernized: 60.22 ± 16.28; balanced: 60.37 ± 17.43), indicating weak direct influence of dietary patterns on mental health and the need for comprehensive analysis with confounding factors like work stress and sleep quality.

### 4.5 Study limitations

Several limitations of the present study should be acknowledged to contextualize the interpretation of its findings. First, the cross-sectional design enables the identification of associations between dietary patterns and quality of life but cannot establish a causal relationship, as it fails to capture temporal sequences of exposure and outcome (26). Second, convenience sampling was adopted for surveying nurses in tertiary hospitals; this method inherently introduces selection bias, as it does not ensure random representation of the target population, thereby restricting the generalizability of the findings to the broader cohort of tertiary hospital nurses. Third, all study data—including dietary intake, body weight, and scores from the 36-Item Short Form Health Survey (SF-36)—were collected via self-reported questionnaires distributed through the online platform “Questionnaire Star.” Such self-reported data are susceptible to recall bias (e.g., inaccurate retrospective memory of dietary consumption details) and social desirability bias (e.g., overreporting healthy behaviors to conform to perceived norms), which may marginally compromise the accuracy of dietary pattern classification. Fourth, factor analysis, used to derive dietary patterns, relies on empirical inference and involves inherent subjectivity in selecting factor rotation methods and defining common factors, which could influence the robustness of the dietary pattern constructs. Additionally, potential confounding variables—including physical activity levels, sleep quality, and workload intensity—that are known to affect both dietary choices and overall quality of life were not fully controlled for in the analysis (27), which may have attenuated or exaggerated the observed associations.

In future research, targeted design optimizations will be implemented to address these limitations: probability sampling methods (e.g., stratified random sampling) can be adopted to enhance sample representativeness and reduce selection bias; objective measurement tools (e.g., 24-h dietary recalls supplemented with food diaries for dietary data, or researcher-conducted anthropometric measurements to replace self-reported body weight) can mitigate self-report-related biases; validated instruments will be integrated to better control for confounding variables and elucidate the independent effect of dietary patterns; and more rigorous approaches (e.g., predefining factor analysis protocols) can be applied to minimize subjectivity in dietary pattern derivation. These adjustments will improve the precision, reliability, and causal inferential power of subsequent studies.

## 5 Conclusion

This study specifically explored the relationship between detailed dietary patterns and multi-dimensional quality of life in a targeted population of night-shift nurses within the context of Chinese tertiary hospitals. Three primary dietary patterns—Traditional, Western, and Balanced—were identified among night-shift nurses in tertiary hospitals in Hangzhou. Night-shift nurses adhering to traditional and balanced dietary patterns demonstrated better quality of life compared to those following the western dietary pattern. These findings underscore the importance of promoting reasonable dietary choices and minimizing adherence to western dietary patterns to support nurses’ health. Based on the data from this study, it is recommended that night shift nurses in tertiary hospitals increase their intake of fruits, vegetables and high-quality protein. Hospitals can assist with dietary intervention through measures such as “nutritional guidance and optimization of night shift meals” to improve the quality of life of nurses. Further research with a larger sample size is needed to verify the conclusions and provide a basis for wider promotion. It is necessary to ensure that all suggestions are supported by data and avoid overgeneralization. It is recommended to conduct in-depth longitudinal research in the future to clarify the causal relationship between dietary patterns and quality of life. Nursing administrators are encouraged to collaborate with relevant hospital departments to adjust food supply models according to night-shift schedules, thereby offering optimized dietary options. Such measures may facilitate the adoption of healthier dietary patterns, contribute to humanistic care, and improve nurses’ sense of fulfillment.

## Data Availability

The original contributions presented in the study are included in the article/supplementary material, further inquiries can be directed to the corresponding author.
